# Equity and elderly health in India: reflections from 75th round National Sample Survey, 2017–18, amidst the COVID-19 pandemic

**DOI:** 10.1186/s12992-020-00619-7

**Published:** 2020-10-08

**Authors:** Alok Ranjan, V. R. Muraleedharan

**Affiliations:** Department of Humanities and Social Sciences, Indian Institute of Technology-Madras, Chennai, India

**Keywords:** Elderly heath, Equity, COVID-19, India

## Abstract

**Background:**

Severe Acute Respiratory Syndrome Coronavirus-2 (SARS-CoV-2) outbreak, called coronavirus disease - 2019 (COVID-19), has affected more than 200 countries across the globe with a higher fatality rate among the elderly population. Aim of the study is to highlight the vulnerability of the aged amidst the current COVID-19 pandemic, and in the light of the recent international evidence, suggests what government could do to mitigate their vulnerability.

**Methods:**

Data from the recently released (November 2019) 75th Round National Sample Survey (NSS), which was conducted from July 2017 to June 2018, across 8077 rural villages and 6181 urban wards was used for this study. Data collected from 555,115 individuals (rural: 325,232; urban: 229,232) included 42,762 elderly individuals (60 years or above). Bivariate and multivariate analyses were used for the calculation.

**Results:**

Of the total sample of elderly individuals, 27.7% reported suffering from an ailment in the last 15 days, whereas 8.5% had been hospitalized during the last 365 days. Among the elderly, hospitalization rate was higher in the urban areas (OR: 1.23), general social category (OR: 1.18), richest economic quintile (OR: 1.69), and among those living alone (OR: 2.40). Also, among the elderly, 64% of those in the scheduled tribe (social group) and 51% in the poorest economic quintile utilized public facilities for hospitalization. Cardiovascular ailments were the major cause for hospitalization (18.1%) and outpatient visit (32%) among the elderly. Ailments related to diabetes and hypertension constituted 55% of outpatient visit for the elderly. Only 18.9% of the elderly had health insurance though chances of facing catastrophic health expenditures were high among the elderly. 6.6% of elderly female and 1.6% male live alone, and 27.5% of age 80 years and above are immobile. 50% of male and 90% of female are financially dependent on others and more so in poorer economic quintiles.

**Conclusions:**

The vulnerability of India’s elderly increases across economic levels, and other dimensions such as the place of residence, gender, social group (caste), marital status, living arrangements, surviving children, and economic dependence. The current COVID-19 pandemic poses a greater risk of social isolation among the elderly, which may cause detrimental health impact.

**Trial registration:**

Not applicable since the study is based on secondary data.

## Background

As the current Coronavirus Disease (COVID-19) pandemic spreads across the globe, the elderly population (60 years and above) become particularly vulnerable [1, 2]. Mortality data from different countries and various studies show that the elderly population is more susceptible compared to their younger counterparts [2, 3]. However, all elderly are not equally vulnerable to COVID-19. Mortality data from South Korea show fatality rates due to COVID-19 were 1.8% in the age group of 60–69 years, 6.3% in 70–79 years, and 13% in the age group 80 years and above [4]. Emerging studies from the United States show that the chances of contracting COVID-19 were three times higher in black counties compared to white counties, and similarly, the death rate in black counties was six times higher than in white counties [5]. Even in India, spread and fatality of COVID-19 are significantly higher in slum areas where poor people live [6].

In this regard, it is imperative to understand the current status of the elderly health and related socioeconomic dimensions in India. This may provide valuable insights about mitigation strategies to take care of the elderly during the current COVID-19 pandemic, equitably. Recently released data (November. 2019) by the National Sample Survey (NSS) for its 75th Round, 2017–18, on social consumption related to health, provides an opportunity to understand the health status of elderly in India [7], and social support systems the elderly have across socioeconomic groups. To the best of our knowledge, this is the most recent national-level unit data on the elderly we have for India, before the outbreak of the COVID-19 pandemic. The primary aim of this study is to present the health status of the elderly in the country across various socioeconomic categories from this national level survey, and to relate it with the potential impact of COVID-19 pandemic. In the elderly population too, vulnerability varies across various dimensions such as place of residence, gender, social group (caste), occupation, income levels, living arrangements, and economic dependence. This study also aims to provide insights into interplay of these factors. This paper is organized as follows: Section II presents details of the methods used for our analysis; Section III presents results related to elderly health status, access to healthcare, financial risk protection, living arrangements, economic dependence, physical immobility, perception of self-health, and changes in the health status of elderly from 2014 to 2017–18; Section IV presents a discussion on lessons learned from these findings and offers a few suggetions for mitigating the adverse impact of the current pandemic on the elderly in India; and Section V offers a few concluding remarks.

## Methods

Data for the current study is extracted from the 75th Round of NSS, which is a nation-wide sample survey conducted by the Government of India from July 2017 to June 2018 [7]. This survey covered 113,823 sample households and 555,115 individuals (Rural: 325,883; Urban: 229,232; Male: 283,200; Female: 271,877) from randomly selected 8077 villages and 6181 urban wards by two-stage random sampling method. In the first stage, rural villages and urban wards were selected, and in the second stage, households were selected. The entire sample included 42,762 elderly individuals.

The 75th Round NSS, 2017–18, collected information related to demographic details, household characteristics, morbidity and mortality, hospitalization in the last 365 days, health insurance coverage, out-of-pocket expenditure (OOPE), healthcare utilization, immunization coverage, maternal health, and elderly health [8]. Survey considerd 15 days recall period in self-reporting of acute ailments. One of the reasons for choosing 15 days recall period over monthly recall (30 days) was to reduce the recall bias in reporting the ailments. There is a higher chance of forgetfulness in monthly reporting compared to 15 days recall. Also, monthly reporting of acute ailment might erase significant fraction of relevant actions (doctors visit, expenditure on health, duration of illness etc.) taken by individuals, and more so in lower socio-economic population [8]. The current study focuses on elderly health and indicators related to this group.

For analysis purpose, age of the elderly groups were categorized as 60–69, 70–79, and 80 and above. Employment status was broadly categorized as self-employed, regular wage, casual labourer, and others. The economic quintiles for the household were assigned based on the Usual Annual per capita Consumption Expenditures (UAPCE) for rural and urban areas, respectively. UAPCE includes household expenditures other than for healthcare. It categorizes households into five economic quintiles (1-poorest, 2-poor, 3-middle, 4-rich, 5-richest). All individuals above the age of seven were categorized under broader education categories of illiterate, up to primary (8th std.), up to secondary (10th std.), and above secondary level.

Members of households were asked whether they were hospitalized in the last 365 days; and whether they suffered from any long term chronic ailment or acute ailment in the last 15 days. Data on chronic and acute ailments were used for calculating Proportion of Ailing Person (PAP) in the last 15 days.

The survey also collected information from households on whether they sought care from private or public facilities, amount of money spent for various services, including physician fees, drugs, diagnostics, and non-medical expenses such as travel, food etc., for both inpatient and outpatient services, and who paid for such expenses.

A total of 63 different reported ailments were broadly grouped under the following 15 ailment-categories. These are: infections, cancer, blood disease, endocrine and metabolic disease including diabetes, psychiatric and neurological, genito-urinary, eye, ear, cardio-vascular including hypertension, respiratory, gastrointestinal, skin, musculoskeletal, injuries, obstetric, and unclassified conditions.

Detailed information on how expenses were met from various sources and who paid for such expenses were obtatined for each episode of illness and service utilization. Out of pocket expenditures (OOPEs) were calculated (which is net of medical and transportation expenditures after deducting reimbursement from the insurance schemes). Catastrophic health expenditure at 10% (CHE-10) and 25% (CHE-25) threshold was calculated if the total annual health expenditure of the household was higher than 10% and 25%, respectively, of UAPCE, based on WHO Sustainable Development Goals (SDGs) [9].

Elderly were asked additional questions about their living conditions: whether 1) Living with spouse and other members, 2) Living with spouse only, 3) Living without spouse but with children or relatives, 4) Living alone but not as an inmate of old age home, and 5) Living alone as an inmate of old age home. Financially, whether they are fully independent, partially independent, or fully dependent on others. For our analysis, partially dependent or fully dependent were considered as “dependent”.

Besides, information on their physical mobility, which is an indicators of their disability [10], was recorded. In present study, “confined to bed”, “confined to home”, “movement on wheelchair” are considered as ‘physically immobile’.

Elderly were also asked about their surviving children. We categorize them as living as “at least one living child” and “no child” for analysis purpose.

It is important to note that the elderly were asked about their self-perception of current health status, which was categorized as excellent, good, and poor. They were also asked to compare their perception of current health status with the previous year’s health status, such as, ‘much better’, ‘somewhat better’, ‘nearly the same’, ‘somewhat worse’, and ‘worse’. “Somewhat worse” and “worse” have been categorized as “worse” in our analysis.

Binary logistic regression was used to understand factors affecting hospitalization, PAP, CHE-10, CHE-25, living arrangements, and economic dependence in the elderly. In case of hospitalization, the dependent variable was the incidence of hospitalization, and independent variables were age group, place of residence, gender, surviving children, social group (caste), education category, household occupation, economic quintile, insurance coverage, economic independence, and living arrangements. Similarly, for the incidence of PAP, the same independent variables were used, except insurance coverage since insurance schemes do not cover outpatient care. For CHE-10 and CHE-25, other than the above mentioned independent variables, choice of provider (public or private) was included in the model. To understand factors affecting living arrangements and economic independence in the elderly population, chances of ‘living alone’, and chances of ‘being dependent’ were dependent variables, with same independent variables (except economic independence, and insurance coverage in living arrangement model; and household occupation, and insurance coverage in financial dependent model) mentioned above for the logit model of hospitalization. These variables have been included based on suggestions from existing literature [11–16]. It is important to note here that the literature also suggests that “marital status” be considered as an explanatory variable. We have dropped this factor as it showed a high multicollinearity (Variation Inflation Factor: 23). No multicollinearity was found among independent variables used in our analysis (refer Additional File 1).

Findings of the 75th Round NSS, 2017–18, were also compared with 71st Round NSS, 2014, for similar indicators, to understand the change in state of elderly health from 2014 to 2017–18. Bivariate and multivariate analyses were performed using STATA version 14.1.

## Results

Findings of the study are presented under eight themes: 1) Demographic characteristics, 2) Disease burden and access to healthcare, 3) Financial hardship, 4) Living arrangements, 5) Economic dependence, 6) Physical immobility, 7) Perception towards own health, and 8) Change in health status of the elderly from 2014 (71st Round NSS) to 2017–18 (75th Round NSS). All observations relate to the elderly population, unless stated otherwise.

### Demographic characteristics

The average age of the elderly population in India was 67.5 years (Table 1). Out of total elderly population, 66.1% are in the age group of 60–69 years, 25.9% in 70–79, and 8% are aged 80 years and above. 67.1% of India’s elderly live in rural areas. Proportion of female (50.9%) is higher than male (49.1%). In terms of the social groups, 6.2% elderly belongs to scheduled tribe (ST) category, 17.4% scheduled caste (SC) category, 42.3% to other backward class (OBC), and 34.3% belongs to general category. In India, 54.1% of the elderly people are illiterate, and 20% belongs to those households where casual labor is the main household occupation. Also, 4.3% of the elderly in India do not have a surviving child (Table 1).

**Table 1:** Demographic and Socio-economic characteristics of sample elderly population in India

	India	Sample size (N)
Mean Age (years)	67.5	42,762
Age group (years)		
60–69	66.1	27,769
70–79	25.9	11,235
80 and above	8.0	3758
Place of Residence		
Rural	67.1	23,599
Urban	32.9	19,163
Gender		
Male	49.1	21,902
Female	50.9	20,858
Marital Status		
Never Married/ divorced/ separated	0.9	395
Currently married	64.7	29,324
Widowed	34.4	13,043
Surviving Children		
At least one surviving child	95.7	41,409
No child	4.3	1353
Social Groups		
ST	6.2	3913
SC	17.4	6133
OBC	42.3	16,519
General	34.3	16,197
Education		
Illiterate	54.1	20,194
Up to primary	21.1	9375
Up to secondary	14.3	7752
Above Secondary	10.5	5441
Household occupation		
Self employed	48.1	20,986
Regular Wages	15.5	8536
Casual Labourer	20.0	6709
Others	16.5	6531
Economic quintile-Rural		
Poorest	19.1	3773
Poor	19.0	3945
Middle	21.0	4651
Rich	19.5	4949
Richest	21.3	6281
Economic quintile-Urban		
Poorest	21.7	4776
Poor	18.0	3614
Middle	20.9	3696
Rich	22.0	3537
Richest	17.3	3540

Source: Authors’ computation from unit records of NSSO 75th Round 2017–18

### Disease-burden and access to healthcare

#### Outpatient care

Out of every 100 elderly, 27.7 pesons reported ailments during the previous 15 days; of this, 22.4 reported chronic ailments, and 5.7 reported acute ailments (Table 2). PAP was significantly high among 80 years and above (36.7%), those in urban areas (34.0%), widowed (30.8%), general category (33.2%), having regular wages (31.5%) and in the richest economic quintile (rural-36.8%, urban-43.8%, Table 2, Table 3). The logistic model shows that chances of reporting ailment in last 15 days was 1.50 times higher among 80 years and above compared to those in age group 60–69 years; 1.44 times higher in urban areas compared to rural areas, 2.08 times higher in general category compared to lower socio-economic groups (ST category), 1.33 times higher among those with primary level education compared to illiterate, 1.23 times higher among casual labourers compared to regular wage earners, and 2.47 times higher among those in the richest economic quintile compared to their poorest counterparts (Table 3).

**Table 2:** Disease burden and access to healthcare in elderly population of India

	Hospitalization (in %)		Out-patient care (in %)			
	Hospitalization rate	Share of hospitalization under public sector	Proportion of population reporting chronic condition	Proportion of population reporting ailment in last 15 days	PAP in last 15 days	Share of PAP under public sector
Total	8.5	39.8	22.4	5.7	27.7	33.6
Age group (years)						
60–69	7.1	40.4	20.3	5.2	25.1	34.0
70–79	10.1	40.8	25.3	6.8	31.7	33.2
80 and above	14.3	35.5	31.2	6.8	36.7	32.2
Place of Residence						
Rural	7.7	44.5	19.0	6.0	24.6	39.7
Urban	10.1	32.7	29.5	5.2	34.0	25.5
Gender						
Male	9.5	40.3	22.2	5.8	27.5	33.5
Female	7.5	39.3	22.6	5.7	27.9	33.7
Marital Status						
Never Married/ divorced/	8.8	41.8	23.1	2.2	25.0	47.6
Currently married	7.8	38.1	21.2	5.3	26.1	32.1
Widowed	7.7	40.4	24.8	6.5	30.8	35.7
Surviving Children						
At least one surviving child	7.8	38.9	22.5	5.8	27.9	33.5
No child	21.3	46.0	19.9	4.5	24.2	37.0
Social Groups						
ST	5.5	64.0	10.6	7.3	17.8	43.5
SC	7.5	53.2	18.4	6.5	24.7	38.6
OBC	8.2	38.4	20.9	5.6	26.0	42.2
General	10.0	33.8	28.5	5.3	33.2	22.7
Education						
Illiterate	6.1	46.1	17.8	5.9	23.4	37.7
Up to primary	10.3	42.8	26.5	6.8	32.6	41.2
Up to secondary	9.7	29.1	30.4	4.9	34.5	26.7
Above Secondary	8.0	17.4	27.5	3.7	30.8	15.3
Household occupation						
Self employed	7.1	36.5	20.1	6.0	25.8	31.0
Regular Wages	9.2	37.4	25.7	6.2	31.5	27.1
Casual Labourer	6.5	53.8	18.6	5.8	24.0	50.3
Economic quintile-Rural						
Poorest	5.0	51.0	9.6	7.3	16.7	45.3
Poor	5.0	52.2	15.8	5.9	21.5	34.9
Middle	6.7	47.1	15.6	6.2	21.6	39.1
Rich	8.3	44.3	20.0	5.6	25.3	39.4
Richest	11.2	37.7	32.7	5.1	36.8	40.5
Economic quintile-Urban						
Poorest	9.7	47.7	22.5	4.7	26.7	41.6
Poor	9.7	41.6	28.4	3.4	31.4	31.5
Middle	9.7	35.1	27.3	8.3	35.1	27.1
Rich	10.1	26.4	30.8	4.8	34.7	22.5
Richest	11.0	14.4	40.2	4.3	43.8	12.5

Source: Authors’ computation from unit records of NSSO 75th Round 2017–18

**Table 3:** Factors affecting hospitalization, PAP, CHE-10/25, living arrangements, and economic dependence in India’s elderly

Total	Reporting of hospitalization OR (95% CI)	Reporting of PAP OR (95% CI)	CHE-10 OR (95% CI)	CHE-25 OR (95% CI)	Living alone OR (95% CI)	Economically dependent OR (95% CI)
**Age group (years, ref:60–69)**						
70–79	1.33 (1.27–1.40)*	1.25 (1.19–1.32)*	1.02 (0.93–1.13)	1.07 (0.96–1.19)	1.02 (0.86–1.21)	1.93 (1.82–2.04)*
80 and above	1.37 (1.27–1.48)*	1.50 (1.39–1.61)*	1.00 (0.87–1.16)	0.99 (0.84–1.16)	0.86 (0.65–1.12)	3.59 (3.23–3.99)*
**Place of Residence (ref: rural)**						
Urban	1.23 (1.16–1.29)*	1.44 (1.37–1.51)*	0.50 (0.45–0.56)*	0.53 (0.47–0.59)*	0.86 (0.72–1.02)	0.98 (0.92–1.03)
**Gender (ref: male)**						
Female	0.69 (0.65–0.72)*	0.98 (0.93–1.03)	0.79 (0.71–0.87)*	0.78 (0.70–0.88)*	2.82 (2.38–3.35)*	10.13 (9.53–10.8)*
**Surviving Children (ref: no child)**						
At least one child	0.97 (0.85–1.09)	1.32 (1.16–1.50)*	0.75 (0.60–0.95)	0.81 (0.64–1.03)	0.24 (0.19–0.30)*	2.06 (1.80–2.36)*
**Social Groups (ref: ST)**						
SC	1.09 (0.99–1.20)	1.81 (1.63–2.02)*	1.35 (1.10–1.66)**	1.55 (1.19–2.00)**	1.28 (0.92–1.79)	1.06 (0.95–1.17)
OBC	1.16 (1.07–1.27)**	1.89 (1.71–2.08)*	1.46 (1.21–1.75)*	1.68 (1.33–2.11)*	1.22 (0.90–1.64)	1.20 (1.09–1.31)*
General	1.18 (1.08–1.29)*	2.08 (1.89–2.29)*	1.41(1.17–1.69)*	1.59 (1.26–2.01)*	1.22 (0.90–1.65)	1.19 (1.08–1.30)*
**Education (ref: illiterate)**						
Up to primary	1.09 (1.03–1.16)**	1.33 (1.26–1.41)*	1.18 (1.05–1.33)**	1.26 (1.11–1.44)*	0.73 (0.60–0.90)**	0.85 (0.79–0.91)*
Up to secondary	0.98 (0.92–1.05)	1.17 (1.10–1.25)*	1.40(1.22–1.60)*	1.50(1.30–1.73)*	0.36 (0.28–0.46)*	0.54 (0.50–0.58)*
Above Secondary	0.91(0.84–0.99)**	1.02 (0.94–1.11)	1.48 (1.26–1.74)*	1.60(1.34–1.90)*	0.34 (0.26–0.45)*	0.26 (0.24–0.28)*
**Household occupation (ref: self-employed)**						
Regular Wages	1.02 (0.95–1.08)	1.12 (1.05–1.19)*	1.01 (0.89–1.14)	0.93 (0.81–1.08)	0.49 (0.28–0.87)**	NA
Casual Labourer	1.03 (0.96–1.10)	1.23 (1.15–1.32)*	0.99 (0.86–1.13)	1.13 (0.97–1.32)	3.01 (2.21–4.09)*	NA
**Economic quintile (ref: poorest)**						
Poor	1.11 (1.03–1.20)**	1.29 (1.19–1.39)*	0.63 (0.53–0.73)*	0.63 (0.53–0.74)*	0.60 (0.45–0.81)**	0.96 (0.88–1.04)
Middle	1.31 (1.22–1.41)*	1.56 (1.44–1.68)*	0.56 (0.48–0.64)*	0.59 (0.50–0.69)*	0.79 (0.61–1.03)	0.90 (0.83–0.97)**
Rich	1.44 (1.38–1.55)*	1.68 (1.56–1.81)*	0.47 (0.40–0.54)*	0.51 (0.43–0.60)*	1.11 (0.87–1.42)	0.80 (0.74–0.87)*
Richest	1.69 (1.56–1.82)*	2.47 (2.29–2.66)*	0.36 (0.31–0.42)*	0.40 (0.34–0.47)*	1.38 (1.09–1.75)	0.71 (0.65–0.77)*
**Insurance coverage (ref: No)**						
Yes	1.27 (1.20–1.34)*	NA	0.52 (0.47–0.58)*	0.57 (0.50–0.64)*	NA	NA
**Provider (ref: public)**						
Private	**NA**	NA	8.18 (7.41–9.02)*	7.52 (6.6–8.51)*	NA	NA
**Economic independence (ref: independent)**						
Dependent	1.39 (1.31–1.47)*	1.26 (1.19–1.33)*	1.06 (0.95–1.18)	0.94 (0.83–1.06)	NA	NA
**Living arrangement (ref: with spouse/family)**						
Living alone	2.40 (2.07–2.78)*	1.66 (1.43–1.93)*	2.07 (1.62–2.64)*	2.04 (1.59–2.62)*	NA	0.16 (0.14–0.19)*
**Constant**	0.16 (0.14–0.19)*	0.05 (0.04–0.06)*	0.56 (0.41–0.76)*	0.14 (0.10–0.19)*	0.009(0.006–0.014)*	0.64 (0.54–0.75)*
**Model Details**						
Log likelihood	−23,767.779	−24,171.993	− 6115.6486	− 5128.930	− 2938.437	−19,561.532
Number of observations	42,755	42,755	10,801	10,801	42,760	42,755
LR Chi2	1369.42	1974.30	2655.74	1793.75	2396.66	12,375.97
Prob>Chi2	0.000	0.000	0.000	0.000	0.000	0.0000
Pseudo R2	0.028	0.039	0.178	0.149	0.2897	0.2403
Mean Variance inflation factor	1.56	1.58	1.65	1.65	1.63	1.620
Mean Pregibon dbeta	0.258	0.275	0.45	0.245	0.019	0.709
Specification error (linktest): predicted value (_hat)[p > |z|]	0.000	0.000	0.000	0000	0.000	0.000
Specification error (linktest): predicted value squared (_hatsq) [p > |z|]	0.315	0.141	0.369	0.314	0.387	0.081

Note:

(*) *p*-value < 0.001

(**) *p*-value< 0.05

‘NA’ indicates particular variable was not included the respective model

All estimates, except model details, are odds ratio (OR) and values in the parentheses are confidence interval of the estimates

Source: Authors’ computation from unit records of NSSO 75th Round 2017–18

Cardiovascular conditions including hypertension (32.0%), endocrine conditions including diabetes (22.5%), musculoskeletal conditions (13.9%), infectious diseases (10.0%), and respiratory ailments (7.3%) were the top-five conditions for seeking outpatient care among the elderly in the last 15 days (Table 4). 33.6% of the elderly went to a public provider in the last 15 days, particularly for cancer (55.8%) and eye-related problems (47.5%,Table 4). Public healthcare utilization was higher in rural areas (39.7%), ST category (43.5%), casual labourer (50.3%), illiterate (37.7%), never married or divorced (47.6%) and poorest economic quintile compared to their respective counterparts (rural-45.3%, urban-41.6%, Table 2).

**Table 4:** Disease burden and health seeking behavior in elderly during hospitalization and out-patient care in India

	Hospitalization		Out-patient care	
	Diseases burden during hospitalization	Share of hospitalization episodes treated under public sector	Diseases burden in out-patient care	Share of out-patient care treated under public sector
Infection	16.6	48.3	10.0	33.5
Cancers	4.6	52.8	0.5	55.8
Blood diseases	0.9	46.8	0.9	15.3
Endocrine, metabolic, nutritional (includes diabetes)	5.3	40.3	22.5	35.5
Psychiatric and Neurological	8.2	36.8	4.4	28.2
Genito-urinary	5.3	27.8	1.0	33.9
Eye	8.4	37.8	1.5	47.5
Ear	0.2	36.4	0.4	35.6
Cardio-vascular (includes hypertension)	18.1	37.7	32.0	33.4
Respiratory	7.8	45.3	7.3	37.5
Gastro-Intestinal	7.5	36.7	2.6	30.3
Skin	0.7	50.3	0.9	22.7
Musculo-skeletal	6.2	34.8	13.9	33.0
Injuries	7.9	34.9	0.7	31.1
Others	2.4	31.4	1.6	15.3
Total	100.0	39.8	100.0	33.6

Source: Authors’ computation from unit records of NSSO 75th Round 2017–18

#### Inpatient care

Overall hospitalization rate among the elderly was 8.5%, and was highest among 80 years and above (14.3%) - it was significantly high for male (9.5%), those in urban areas (10.1%), never married/divorced (8.8%), those with no surviving children (21.3%), in general social category (10.0%), regular wages earners (9.2%), and those in the richest economic quintile (rural-11.2%, urban-11%-Table 2 and Table 3). Cardiovascular diseases (18.1%), infectious diseases (16.6%), eye ailments (8.4%), psychiatric or neurological conditions (8.2%), and injuries (7.9%) were the top five reasons for hospitalization in the last 365 days (Table 4).

Public facilities accounted for 39.8% of all inpatient services availed by the elderly - it was higher in rural areas (44.5%), those with no surviving children (46.0%), ST category (64.0%), illiterate (46.1%), and poorest income quintile compared to respective counterparts (rural-51.0%, urban-47.7%, Table 2). Share of the public sector was higher for cancer treatment (52.8%), skin related ailments (50.3%), infectious diseases (48.3%), and blood diseases (46.8%) whereas the share of the private sector was higher for most other conditions. For instance, private facilities accounted for 72.2% of genito-urinary, 63.2% of psychiatric and neurological conditions, and 63.1% of injury related inpatient care (Table 4).

### Financial risk protection

Publicly funded health insurance (PFHI) coverage and (tax funded) subsidized public provisioning are the two major strategies used by the government for providing financial risk protection in India [11].

#### Health insurance

Overall, 18.9% of the elderly were coved under health insurance schemes; whereas PFHIs covered 14.3% population. PFHIs only cover inpatient care in India, whereas Central Government Health Schemes (CGHS-2.1%) and ESIS (0.7%) cover outpatient care as well. Private insurance also provided coverage (1.8%), but for inpatient care alone [7]. PFHIs coverage among the elderly was high in rural areas (16.6%), ST category (20.7%), illiterate (16.6%), casual labourer (18.2%), and poorest rural quintile (12.8%) in India. Insurance coverage in urban areas was more equitable compared to rural areas, since PFHI coverage was higher in poorer quintile compared to richer quintile in urban elderly. In the rural areas, PFHI coverage was higher in top two quintiles compared to the bottom two quintiles (Table 5).

**Table 5:** Financial Protection during hospitalization and outpatient care for India’s Elderly

	Insurance coverage		OOPE in out-patient care		OOPE during hospitalization		CHE-10		CHE-25	
	Total insurance coverage	Coverage under PFHI	Pub	Pvt	Pub	Pvt.	Pub	Pvt	Pub	Pvt.
Total	18.9	14.3	390	852	6209	38,709	23.2	64.9	9.1	37.0
Age group (years)										
60–69	19.3	14.5	410	822	5315	39,051	22.2	67.5	7.6	38.8
70–79	17.8	13.9	336	841	8364	38,523	25.4	63.0	13.0	36.6
80 and above	18.7	14.3	430	1039	4745	37,828	23.0	58.9	6.4	30.7
Place of Residence										
Rural	18.1	16.6	388	816	6180	32,009	25.2	67.8	10.0	40.7
Urban	20.4	9.5	394	892	6268	47,200	19.0	61.3	7.1	32.4
Gender										
Male	19.0	14.1	441	857	7336	44,666	25.7	67.6	10.8	41.2
Female	18.7	14.5	342	847	4780	31,459	20.2	61.7	6.9	31.9
Marital Status										
Never Married	17.3	14.6	318	1849	7857	88,010	16.6	62.0	5.7	46.6
Currently married	18.3	13.5	484	861	6381	40,639	21.7	67.1	9.5	38.6
Widowed	19.9	15.8	256	818	4370	26,638	19.8	58.1	6.1	29.7
Surviving Children										
At least one surviving child	18.7	14.1	393	849	5676	36,426	20.3	63.5	8.1	35.3
No child	21.3	18.9	306	937	9320	56,542	40.6	77.0	14.8	51.6
Social Groups										
ST	22.5	20.7	246	613	4102	22,546	25.2	69.2	3.5	43.3
SC	15.1	13.6	451	1063	7229	24,972	27.8	61.2	12.8	31.8
OBC	20.0	16.9	324	782	5523	33,098	23.6	66.8	8.9	38.8
General	18.8	10.3	492	860	6779	48,538	19.9	64.1	8.5	36.5
Education										
Illiterate	17.9	16.6	333	732	4409	25,878	19.7	63.5	6.4	34.3
Up to primary	19.3	14.7	363	856	6065	31,672	21.9	65.2	11.6	33.9
Up to secondary	19.4	11.8	530	786	6678	46,869	23.7	63.7	8.7	39.6
Above Secondary	22.2	4.8	615	1222	13,856	57,350	21.2	63.3	7.5	36.6
Household occupation										
Self employed	14.9	13.0	500	846	6961	37,673	19.7	63.5	9.4	35.9
Regular Wages	24.3	10.4	308	1046	5998	36,478	21.9	65.2	5.3	26.3
Casual Labourer	18.8	18.2	268	618	3509	22,085	23.7	63.7	8.2	36.0
Others	25.2	17.1	387	830	5260	42,098	21.2	63.3	8.4	43.3
Economic quintile-Rural										
Poorest	13.5	12.8	381	761	5084	19,410	43.3	75.2	17.5	47.4
Poor	10.6	10.1	477	908	4858	28,978	24.3	67.7	4.4	45.1
Middle	19.0	18.2	360	725	6293	24,807	19.3	71.4	8.7	37.8
Rich	20.9	19.6	421	659	5470	32,435	22.6	72.9	10.7	44.6
Richest	25.6	21.7	353	918	7949	39,683	22.7	60.6	9.9	36.5
Economic quintile-Urban										
Poorest	13.4	10.4	371	995	5577	32,077	27.6	77.1	11.8	44.4
Poor	18.1	13.2	322	862	4227	41,811	21.6	63.2	6.4	34.7
Middle	18.3	9.1	352	845	5850	42,262	13.6	63.8	3.7	34.2
Rich	19.2	9.0	441	868	7091	50,633	11.6	62.3	4.9	28.2
Richest	35.6	5.5	564	916	13,025	60,067	11.2	48.6	6.2	26.0

Source: Authors’ computation from unit records of NSSO 75th Round 2017–18

#### Outpatient care

OOPE for outpatient care was Rs. 390 per visit under public sector, and Rs.852 per visit under private sector (Table 5). OOPE was significantly higher for those 80 years and above (public: 430, private: 1039). OOPE was almost the same under rural and urban India. OOPE was higher for male compared to the female gender in the public sector, whereas under the private sector it was nearly the same. OOPE was higher in ST category population compared to general category population in both public and private sectors. The public sector was more equitable compared to the private sector. For instance, under public sector, OOPE was Rs. 371 for the poorest income quintile as Rs.564 for the richest quintile. On the other hand, under the private sector, OOPE was Rs. 995 for the poorest quintile and Rs. 916 for the richest quintile.

#### Inpatient care

Average OOPE was Rs. 6209 under public sector and Rs. 38,709 under private sector. OOPE was significantly higher for male, urban areas, never married or divorced, elderly without children, ST category, above secondary literate, and richest quintiles compared to their respective counterparts. For instance, OOPE for the poorest rural quintile was Rs. 5084 in public and Rs. 19,410 in private provider; whereas it was Rs. 7949 and Rs. 39,683 in the richest rural quintile, respectively (refer Table 5). CHE-10 and CHE-25 were calculated to estimate the impact of OOPE on the households. 23.2% of inpatients in public sector faced CHE-10, whereas 64.9% faced CHE-10 under private sector. Similarly, CHE-25 was 9.1% under public sector, and 37.0% under private sector. CHE-10 and CHE-25 were higher among those in 60–69 years age group, rural areas, male gender, never-married individuals, ST category, casual labourer, and poorest income quintile compared to their counterparts (Table 5). Chances of facing CHE-10 and CHE-25 were statistically higher for rural areas, male gender, those with no surviving children, in the poorest quintile, non-insured population, who used private provider and among the elderly living alone (Table 3). Similarly, chances of facing CHE-10 and CHE-25 was 8.18 and 7.52 times higher, respectively, under the private sector compared to the public sector.

### Living arrangements

4.2% of the elderly population lived alone, whereas 14.1% lived with spouse only (Table 6). The elderly population living alone was higher in rural areas (4.4%), female gender (6.6%), never married or divorced individuals (22.2%), elderly with no surviving child (16.1%), illiterate population (5.0%), and the richest income quintile (rural-7.0%, urban-6.5%). Also, those living with their spouse were higher in the top two income quintiles compared to the bottom two quintiles. Chances of living alone was higher in female (OR: 2.82), and richest income quintile (OR: 1.87- refer Table 3).

**Table 6:** Living arrangement and economic independence in elderly population of India

	Living arrangements				Economic independence			Physically immobile	Poor perception of current health	Perception of change in state of health being worse
	Living with spouse and other members	Living with spouse only	Living without-spouse but with children/relatives	Living alone	Independent	Partially dependent	Fully dependent			
Total	50.3	14.1	31.5	4.2	30.1	22.9	47.0	7.6	19.6	21.0
Age group (years)										
60–69	54.9	16.0	25.1	4.1	35.0	23.9	41.2	4.5	13.6	16.2
70–79	44.2	11.5	39.9	4.5	23.1	22.5	54.4	9.3	26.8	26.3
80 and above	31.7	7.3	57.1	4.0	12.3	17.0	70.8	27.5	46.0	43.7
Place of Residence										
Rural	51.1	13.4	31.1	4.4	28.5	24.5	47.0	7.6	21.4	22.8
Urban	48.6	15.5	32.2	3.7	33.3	19.7	47.0	7.5	16.1	17.3
Gender										
Male	64.1	17.9	16.4	1.6	50.9	22.9	26.1	6.2	17.4	19.6
Female	37.0	10.4	46.0	6.6	10.0	23.0	67.1	8.9	21.7	22.4
Marital Status										
Never Married/	4.6	3.6	69.6	22.2	37.3	17.1	45.6	8.4	21.7	19.4
Currently married	74.8	21.7	3.3	0.3	38.3	23.1	38.6	5.3	16.2	18.6
Widowed	5.4	0.2	83.4	11.0	14.5	22.7	62.8	11.9	26.0	25.6
Surviving Children										
At least one child	52.1	12.8	31.5	3.6	29.1	23.5	47.5	7.6	19.7	21.3
No child	8.9	43.5	31.4	16.1	53.1	11.3	35.6	7.4	17.3	15.0
Social Groups										
ST	48.7	12.6	34.5	4.2	25.7	25.3	49.1	6.4	17.6	15.7
SC	51.1	14.4	30.2	4.2	29.0	25.2	45.8	7.6	21.3	22.6
OBC	48.3	13.1	34.3	4.3	28.5	23.8	47.7	8.0	18.8	20.8
General	52.6	15.4	28.0	4.0	33.4	20.3	46.3	7.2	20.2	21.3
Education										
Illiterate	44.9	11.3	38.8	5.0	21.0	23.5	55.5	8.7	21.8	23.5
Up to primary	54.0	13.2	29.4	3.4	29.5	26.0	44.5	7.6	20.2	20.3
Up to secondary	60.1	17.8	19.8	2.4	42.0	22.2	35.8	5.4	16.9	18.5
Above Secondary	57.0	25.4	13.8	3.8	61.8	15.0	23.2	4.5	10.9	12.8
Household occupation										
Self employed	58.0	9.2	32.1	0.8	30.3	22.6	47.1	7.3	18.9	21.1
Regular Wages	56.3	3.5	39.7	0.5	21.8	26.0	52.2	9.3	17.5	18.4
Casual Labourer	48.3	10.2	38.5	3.1	26.7	25.9	47.4	7.9	22.0	22.8
Economic quintile-Rural										
Poorest	55.3	10.5	29.7	4.5	26.2	28.1	45.7	8.4	21.8	22.5
Poor	54.2	6.0	36.7	3.1	25.4	25.3	49.3	7.6	22.3	20.9
Middle	53.7	12.3	31.5	2.5	27.0	25.7	47.4	6.4	19.5	24.5
Rich	49.2	14.5	31.4	4.8	29.1	23.5	47.4	7.2	21.3	21.8
Richest	43.6	22.7	26.7	7.0	34.2	20.5	45.3	8.3	22.1	24.1
Economic quintile-Urban										
Poorest	54.2	6.6	35.9	3.2	26.3	25.4	48.4	8.5	16.5	20.4
Poor	56.0	7.5	34.3	2.3	30.6	19.5	49.9	7.3	16.9	17.9
Middle	50.4	12.6	34.7	2.3	30.6	18.5	50.8	5.9	17.4	17.3
Rich	45.8	22.2	27.8	4.3	35.9	18.6	45.5	6.8	14.1	15.1
Richest	35.6	29.8	28.2	6.5	45.1	15.7	39.3	9.5	15.5	15.6

Source: Authors’ computation from unit records of NSSO 75th Round 2017–18

### Economic dependence

In India, 47% of elderly were financially depenedent on others; 30.1% were independent, and 22.9% were partially dependent (Table 6). In other words, 70% of India’s elderly were, partially or entirely, financially dependent on others. Complete financial dependence was higher among those 80 years and above (70.8%), female (67.1%), widowed (62.8%), illiterate (55.5%), and poorer quintiles. Chances of being economically dependent were higher among 80 years and above (OR: 3.59), female (OR: 10.13), general category population (OR: 1.19), elderly not living alone, and poorer quintiles (refer Table 3).

### Physical immobility

Physical mobility is one of the proxy indicators for locomotor disability. In India, 7.6% of the elderly were either completely (bedridden) or partially immobile (on a wheelchair or restricted within their home). It was considerably high among those 80 years and above (27.5%), female gender (8.9%), widowed (11.9%), illiterate population (8.7%), and poorer income quintiles. However, immobility increases steeply for the richest quintile (rural-8.3%, urban- 9.5%) compared to other quintiles (refer Table 6).

### Perception of self-health

In India, one in five elderly (19.6%) felt their current health status was poorer, and a similar proportion (21.0%) felt that their health condition had deteriorated compared to the previous year (Table 6). Perception of health being poor was high among those above 80 of years, in rural areas (21.4%), and among the widowed elderly (26%). About 20% of those in poorest urban quintile perceived their health had deteriorated compared to the previous year (refer Table 6).

### Change in the health status of the elderly in NSS 75th round, 2017–18, compared to NSS 71st round, 2014

Hospitalization rate among elderly fell from 10.9% in 2014 to 8.5% in 2017–18 (Table 7). Also, PAP fell from 30.3 (out of 100 reported elder persons) in 2014 to 27.7 in 2017–18. Share of the public sector in outpatient care increased from 28.3% in 2014, to 33.6% in 2017–18, whereas its share in inpatient care increased from 35.9% to 39.0%. OOPEs under public sector fell from Rs. 547 (in 2014) to Rs. 390 (in 2017–18) per visit for outpatient care and from Rs. 7177 (in 2014) to Rs 6209 (in 2017–18) per visit for hospitalization. On the other hand, OOPEs under private sector increased from Rs. 802 (in 2014) to Rs 852 (in 2017–18) per outpatient visit, and Rs. 31,875 (in 2014) to Rs. 38,709 (in 2017–18) per hospitalization visit(refer Table 7).

**Table 7:** Variation in various indicators of elderly health from 71st Round NSS, 2014, to 75th Round NSS, 2017–18

		71st Round NSS, 2014	75th Round NSS, 2017–18
**Access**			
Inpatient care	Hospitalization rate	10.9	8.5
	Share of public sector during hospitalization	35.9	39.0
	Share of private sector during hospitalization	64.1	61.0
Out-patient care	PAP	30.3	27.7
	Share of public sector in out-patient care	28.3	33.6
	Share of private sector in out-patient care	71.7	66.4
**Financial Protection**			
Inpatient care	Any insurance coverage	19.0	18.9
	PFHI coverage	16.2	14.3
	OOPE during hospitalization in public sector (in Rs.)	7177	6209
	OOPE during hospitalization in private sector (in Rs.)	31,875	38,709
	CHE-10 in public sector during hospitalization	27.3	23.2
	CHE-10 in private sector during hospitalization	65.9	64.9
	CHE-25in public sector during hospitalization	12.5	9.1
	CHE-25in private sector during hospitalization	38.2	37.0
Out-patient care	OOPE in out-patient care under public sector (in Rs.)	547	390
	OOPE in out-patient care under private sector (in Rs.)	802	852
**Living Arrangements**			
Living with spouse and other members		47.0	50.3
Living with spouse only		14.8	14.1
Living without spouse but with children/relatives		34.1	31.5
Living alone but not in old age home		4.1	4.2
**Economic Independence**			
Independent		28.3	30.1
Partially independent		20.0	22.9
Totally dependent		51.7	47.0
**Physical mobility**			
Physically immobile-Confine to bed		1.6	1.4
Confined to home		6.0	5.5
Able to move outside but with wheelchair only		0.4	0.6
Physically mobile		92.0	92.5
**Perception towards health**			
Own perception of current state of health			
Excellent/ very good		6.8	8.8
Good/fair		70.6	71.6
Poor		22.4	19.6
Own perception about change in the state of health compared to previous year			
Much better		4.6	5.8
Somewhat better		12.9	12.6
Nearly the same		57.5	60.6
Somewhat worse		20.5	18.3
Worse		4.5	2.6

Source: Authors’ computation from unit records of NSSO 71st, 2014, and 75th Round 2017–18

## Discussion

Here we highlight some key findings and discuss the extent to which the elderly population is vulnerable in the light of the emerging international literature and evidence.

Disease burden in the elderly population (PAP: 27.7%, hospitalization rate: 8.5%- Table 2) is disproportionately higher compared to the population below the age of 60 years (PAP: 5.9%, hospitalization rate: 2.4%) [7]. Self-reported hospitalization rate and PAP were significantly higher in upper socioeconomic population compared to the lower socioeconomic population since perceived healthcare needs are higher in upper socioeconomic population. Various studies have shown that the poor and marginalized sections of society have a higher burden of non-communicable diseases and chronic conditions [17]. In current COVID-19 pandemic, studies from China, Italy, Spain, and the United States have shown that NCD patienst are at higher risk of mortality due to COVID-19 [18–20]. In the elderly population, these comorbid conditions further aggravate the COVID-19 condition and increase mortality [21].

In India, one-third of the elderly went to public healthcare facilities (hospitalization: 39.8%, outpatient care: 33.6% -Table 2); the remaining two-third went to private healthcare facilities. Often, elderly patients require lifelong treatment, curative and rehabilitative, for their chronic conditions. These conditions require regular follow-up, which includes doctor’s consultation, continued medication, and diagnostic tests. India’s healthcare systems, both public and private, do not provide the required level of continuity of care, which leads to poor quality of care for the elderly [22]. Rehabilitative care is almost absent in India’s public health care system and in some urban areas where private sector provides this care is prohibitively costly, which poor and middle income elderly cannot afford [23]. All these may lead to further suffering, poorer quality of life, and mortality for the elderly.

In the current COVID-19 pandemic, the public healthcare system has been overstretched in handling the avalanche of COVID-19 patients. It has seriously disrupted provision of regular services at public healthcare facilities which include immunization, child and maternal health, dialysis services, emergency surgeries, and general outpatient care [24]. A recent study done by Stop TB Partnership shows that for every month of lockdown in India, there would be an additional 2,32,665 tuberculosis (TB) cases and 71,290 deaths in the period of 2020–2025 [25]. Public facilities are major service providers for the poor and marginalized elderly in society. For instance, 64% of ST, 54% of the casual labourer, and 51% of rural poorest elderly took inpatient care under the public facility in 2017–18 (refer Table 2).

Private sector, which provides two-thirds of care for the elderly, has not been able to respond adequately in the country [26, 27]. A significant proportion of private providers has either stopped providing care due to fear of the spread of the diseases or have started charging exorbitantly high which cannot be afforded by the poor and middle-class person of the society [28, 29].

Availability of regular drugs, related to chronic conditions, face additional logistic constraints after the nation-wide lockdown [30]. As part of the latest controversy on the use of hydroxychloroquine drug for covid-19 patients, this drug became unavailable for rheumatic patients, which is a common ailment in the elderly population in India and across the world [31, 32]. Public health facilities, where these drugs are provided free of cost to patients (who are largely poor), reported shortage of regular NCD drugs [33].

Elderly face greater risk of financial hardships due to chronic nature of ailment and comorbidities, which require long term care. Another dimension of financial hardship for the elderly comes from the fact that 70% are partially or wholly financially dependent on others (refer Table 6). It is even higher for those in the lower socioeconomic groups. For instance, 50% of elderly males and 90% of females are dependent on other family members for financial support. In rural areas, elderly parents are dependent on their children who work as a migrant labourer in urban settings. In the current crisis with lockdown, the unemployed, migrants from urban areas to rural areas, would find it difficult to support their families and the elderly dependent parents [34].

One of the direct impacts of the current pandemic for the elderly could be lack of access to food leading to starvation. For instance, a study done by Pradhan shows that 50% of India’s rural households are consuming less food compared to pre-COVID-19 outbreak [35]. Studies across the world also point out that food insecurity, hunger, and malnutrition could beome worse during the pandemic [36].

In terms of social welfare schemes, central and state governments have announced various measures for delivering free food grains and direct transfer of pension for the elderly people [37]. However, there is considerable variations in old age pension across states of India. At all India level, only 29.6% of the elderly receive an old-age pension of the total older population [38]. Evidently, current COVID-19 situation makes the elderly more vulnerable financially.

Physical/social distancing has considerably increased social isolation and more so for the elderly population [39]. Studies have established that social isolation increases depression, suicidality, and a higher chance of increased inflammatory response in the elderly [40, 41]. This will also exacerbate the vulnerability of the elderly who are already suffering from psychiatric or neurological conditions (8.2% of total hospitalization in the last 365 days and 4.4% of PAP in last 15 days). For instance, recent studies published post-COVID-19 outbreak has shown that this pandemic has considerably increased the vulnerability of demented patients and their caregivers across the world [42, 43]. One of the suggestions which have been given to elderly by Ministry of Health and Family Welfare (MoHFW), Government of India [44], and Centre for Diseases Control and Prevention (CDC), Atlanta, United States [45], is to be in contact with significant others in current COVID-19 outbreak, through mobile phone and video call. However, a significant proportion of the elderly population finds it difficult to operate a mobile phone which has become one of the essential skills required for daily living [46]. In India, 4.2% (50 lakhs) of elderly live alone, and it was more so for women (6.6%), never married elderly (22.2%), elderly without children (16.1%) and illiterate (5.0%).

Among the elderly, 7.6% are immobile, and it is even more so among the poorest (rural: 8.4%, urban: 8.5%), illiterate (8.7%), and widowed (11.3%). Often, they are taken care of by a family member; care provision through a hired provider is rare. In current social distancing measures, it has adversely impacted the caregiving for these elderly. Studies also show that family members (as caregivers) have been associated with an elevated level of depression and anxiety, higher use of psychoactive medication, poorer self-reported physical health, compromised immune function, and increased risk of early death [47].

This unprecedented situation of COVID-19 draws our attention towards the need for strengthening public healthcare facilities in the country. The public healthcare system is largely managing the pandemic across the country, despite several weaknesses. COVID-19 is a wake-up call for greater investment in public health facilities which include strengthening public infrastructure, skill building for health professionals, strengthening diseases surveillance system, improving quality of care in public healthcare facilities, and better continuity of care between primary and tertiary care. The idea of “Health and Wellness Centre (HWCs)” under Ayushman Bharat is a welcome decision since it proposes to provide comprehensive primary healthcare at health sub-centre (HSC), or nearer home. The elderly population and poor will be the major beneficiaries of this scheme since they bear a higher burden of diseases and ill-health. Current COVID-19 pandemic shows the need of implementing the HWCs scheme [48].

One of the limitations of this study is that it uses 2017–18 data and not the real-time data collected during the COVID-19 outbreak. Though the national sample surveys throw considerable light on the vulnerability of the elderly, we need a deeper understanding of their lived experiences and coping mechanisms during the covid-19 outbreak. Such studies would provide deeper sociological insights required for more “responsive” policy measures to enhance the quality of life of the elderly.

## Conclusions

The current COVID-19 pandemic poses a greater risk of social isolation among the elderly, which may lead to greater adverse health impact. The poor among the elderly has suffered more than others. As a result, their access to regular primary healthcare services, and continuity of care that is essential for those suffering from non-communicable diseases, given their dependency and lack of mobility, may have worsened further during this pandemic. Overall, given the evidence on the possible hardships that the elderly may be have already gone through during the pandemic and hardships that they may face in the future, the importance of strengthening public health care system cannot be over-emphasized. We urge a much greater investment by government to mitigate adverse impact of the pandemic and enhance the quality of life of the elderly in the future.

## Supplementary information

**Additional file 1: Table S1.** Factors affecting hospitalization in India’s elderly, 2017–18. **Table S2.** Factors affecting PAP in India’s elderly, 2017–18. **Table S3.** Factors affecting catastrophic health expenditure at 10% threshold (CHE-10) in India’s elderly, 2017–18. **Table S4.** Factors affecting catastrophic health expenditure at 25% threshold (CHE-25) in India’s elderly, 2017–18. **Table S5.** Factors affecting ‘living alone’ in India’s elderly, 2017–18. **Table S6.** Factors affecting economic dependence in India’s elderly, 2017–18.

## Data Availability

The present study is based on India’s National Sample Survey, 2017–18, which is freely available in the public domain (http://www.mospi.gov.in/unit-level-data-report-nss-75th-round-july-2017-june-2018-schedule-250social-consumption-health) [7].

## Abbreviations

CHE: Catastrophic health expenditure
CHE-10: Proportion of households in a population who face catastrophic health expenditure computed using the threshold of 10% of usual annual consumption expenditure
CHE-25: Proportion of households in a population who face catastrophic health expenditure computed using the threshold of 25% of usual annual consumption expenditure.
HSC: Health Sub Centre
HWC: Health and Wellness Centre
NSS: National sample survey
NSSO: National sample survey office
OOPE: Out-of-pocket expenditure
PFHI: Public Funded Health Insurance
PHC: Primary Health Centre
UAPCE: Usual annual per capita expenditure
UMPCE: Usual monthly per capita expenditure
