# Supplementary material for: Equity and elderly health in India: reflections from 75th round National Sample Survey, 2017–18, amidst the COVID-19 pandemic
Source: Global Health. 2020 Oct 8;16:93. doi: 10.1186/s12992-020-00619-7 (PMC7542572; doi:10.1186/s12992-020-00619-7)
Supplement: Supplementary file 1 — Additional file 1: Table S1. Factors affecting hospitalization in India’s elderly, 2017–18. Table S2. Factors affecting PAP in India’s elderly, 2017–18. Table S3. Factors affecting catastrophic health expenditure at 10% threshold (CHE-10) in India’s elderly, 2017–18. Table S4. Factors affecting catastrophic health expenditure at 25% threshold (CHE-25) in India’s elderly, 2017–18. Table S5. Factors affecting ‘living alone’ in India’s elderly, 2017–18. Table S6. Factors affecting economic dependence in India’s elderly, 2017–18. [file 12992_2020_619_MOESM1_ESM.docx]

**Additional Table 1: Factors affecting hospitalization in India’s elderly, 2017-18.**

| Total | Odds Ratio | Std. Err. | z | p>z | 95% Conf. Interval | | VIF |
| --- | --- | --- | --- | --- | --- | --- | --- |
| **Age group (years, ref:60-69)** |  |  |  |  |  |  |  |
| 70-79 | 1.330 | 0.034 | 11.120 | 0.000 | 1.265 | 1.398 | 1.060 |
| 80 and above | 1.369 | 0.053 | 8.040 | 0.000 | 1.268 | 1.477 | 1.070 |
| **Place of Residence (ref: rural)** |  |  |  |  |  |  |  |
| Urban | 1.227 | 0.033 | 7.650 | 0.000 | 1.164 | 1.293 | 1.400 |
| **Gender (ref: male)** |  |  |  |  |  |  |  |
| Female | 0.688 | 0.018 | -14.390 | 0.000 | 0.654 | 0.724 | 1.360 |
| **Surviving Children (ref: no child)** |  |  |  |  |  |  |  |
| At least one child | 0.965 | 0.061 | -0.560 | 0.578 | 0.853 | 1.093 | 1.030 |
| **Social Groups (ref: ST)** |  |  |  |  |  |  |  |
| SC | 1.089 | 0.054 | 1.710 | 0.087 | 0.988 | 1.202 | 2.240 |
| OBC | 1.163 | 0.050 | 3.470 | 0.001 | 1.068 | 1.266 | 3.260 |
| General | 1.178 | 0.052 | 3.690 | 0.000 | 1.080 | 1.285 | 3.440 |
| **Education (ref: illiterate)** |  |  |  |  |  |  |  |
| Up to primary | 1.091 | 0.033 | 2.900 | 0.004 | 1.028 | 1.157 | 1.240 |
| Up to secondary | 0.980 | 0.034 | -0.570 | 0.568 | 0.916 | 1.049 | 1.430 |
| Above Secondary | 0.913 | 0.039 | -2.110 | 0.035 | 0.839 | 0.993 | 1.690 |
| **Household occupation (ref: self-employed)** |  |  |  |  |  |  |  |
| Regular Wages | 1.015 | 0.032 | 0.470 | 0.638 | 0.954 | 1.079 | 1.250 |
| Casual Labourer | 1.031 | 0.036 | 0.890 | 0.373 | 0.964 | 1.104 | 1.180 |
| **Economic quintile (ref: poorest)** |  |  |  |  |  |  |  |
| Poor | 1.107 | 0.043 | 2.600 | 0.009 | 1.025 | 1.195 | 1.570 |
| Middle | 1.314 | 0.049 | 7.260 | 0.000 | 1.220 | 1.414 | 1.650 |
| Rich | 1.440 | 0.054 | 9.640 | 0.000 | 1.337 | 1.551 | 1.720 |
| Richest | 1.685 | 0.065 | 13.630 | 0.000 | 1.564 | 1.817 | 2.010 |
| **Insurance coverage (ref: No)** |  |  |  |  |  |  |  |
| Yes | 1.266 | 0.034 | 8.660 | 0.000 | 1.200 | 1.335 | 1.040 |
| **Economic independence (ref: independent)** |  |  |  |  |  |  |  |
| Dependent | 1.389 | 0.040 | 11.350 | 0.000 | 1.312 | 1.470 | 1.360 |
| **Living arrangement (ref: with spouse/family)** |  |  |  |  |  |  |  |
| Living alone | 2.399 | 0.181 | 11.620 | 0.000 | 2.070 | 2.780 | 1.100 |
| **Constant** | 0.159 | 0.013 | -22.630 | 0.000 | 0.136 | 0.187 |  |
| **Model Details** | | | | | | | |
| Log likelihood | -23767.779 | | | | | | |
| Number of observations | 42755 | | | | | | |
| LR Chi2 (22) | 1369.42 | | | | | | |
| Prob>Chi2 | 0.000 | | | | | | |
| Pseudo R2 | 0.028 | | | | | | |
| Mean Variance inflation factor | 1.56 | | | | | | |
| Mean Pregibon dbeta | 0.258 | | | | | | |
| Specification error (linktest): predicted value (_hat)[p>\|z\|] | 0.000 | | | | | | |
| Specification error (linktest): predicted value squared (_hatsq) [p>\|z\|] | 0.315 | | | | | | |

Source: Authors’ computation from unit records of NSSO 75^th^ Round 2017-18

**Additional Table 2: Factors affecting PAP in India’s elderly, 2017-18.**

| Total | Odds Ratio | Std. Err. | z | p>z | 95% Conf. Interval | | VIF |
| --- | --- | --- | --- | --- | --- | --- | --- |
| **Age group (years, ref:60-69)** |  |  |  |  |  |  |  |
| 70-79 | 1.251 | 0.032 | 8.790 | 0.000 | 1.190 | 1.316 | 1.06 |
| 80 and above | 1.495 | 0.057 | 10.540 | 0.000 | 1.388 | 1.612 | 1.07 |
| **Place of Residence (ref: rural)** |  |  |  |  |  |  |  |
| Urban | 1.438 | 0.038 | 13.810 | 0.000 | 1.366 | 1.514 | 1.39 |
| **Gender (ref: male)** |  |  |  |  |  |  |  |
| Female | 0.978 | 0.025 | -0.860 | 0.392 | 0.930 | 1.029 | 1.36 |
| **Surviving Children (ref: no child)** |  |  |  |  |  |  |  |
| At least one child | 1.318 | 0.088 | 4.150 | 0.000 | 1.157 | 1.501 | 1.03 |
| **Social Groups (ref: ST)** |  |  |  |  |  |  |  |
| SC | 1.813 | 0.099 | 10.900 | 0.000 | 1.629 | 2.018 | 2.23 |
| OBC | 1.886 | 0.092 | 13.000 | 0.000 | 1.714 | 2.076 | 3.25 |
| General | 2.081 | 0.103 | 14.860 | 0.000 | 1.889 | 2.293 | 3.42 |
| **Education (ref: illiterate)** |  |  |  |  |  |  |  |
| Up to primary | 1.329 | 0.039 | 9.660 | 0.000 | 1.255 | 1.408 | 1.23 |
| Up to secondary | 1.172 | 0.040 | 4.670 | 0.000 | 1.097 | 1.253 | 1.43 |
| Above Secondary | 1.024 | 0.043 | 0.550 | 0.580 | 0.942 | 1.112 | 1.68 |
| **Household occupation (ref: self-employed)** |  |  |  |  |  |  |  |
| Regular Wages | 1.118 | 0.034 | 3.700 | 0.000 | 1.054 | 1.187 | 1.24 |
| Casual Labourer | 1.233 | 0.042 | 6.170 | 0.000 | 1.154 | 1.318 | 1.18 |
| **Economic quintile (ref: poorest)** |  |  |  |  |  |  |  |
| Poor | 1.289 | 0.050 | 6.470 | 0.000 | 1.193 | 1.391 | 1.57 |
| Middle | 1.556 | 0.059 | 11.670 | 0.000 | 1.444 | 1.675 | 1.64 |
| Rich | 1.682 | 0.064 | 13.650 | 0.000 | 1.561 | 1.812 | 1.71 |
| Richest | 2.465 | 0.094 | 23.690 | 0.000 | 2.288 | 2.656 | 1.98 |
| **Economic independence (ref: independent)** |  |  |  |  |  |  |  |
| Dependent | 1.256 | 0.036 | 7.900 | 0.000 | 1.187 | 1.329 | 1.36 |
| **Living arrangement (ref: with spouse/family)** |  |  |  |  |  |  |  |
| Living alone | 1.663 | 0.128 | 6.590 | 0.000 | 1.430 | 1.934 | 1.1 |
| **Constant** | 0.053 | 0.005 | -33.580 | 0.000 | 0.044 | 0.063 |  |
| **Model Details** | | | | | | | |
| Log likelihood | -24171.993 | | | | | | |
| Number of observations | 42755 | | | | | | |
| LR Chi2 (21) | 1974.30 | | | | | | |
| Prob>Chi2 | 0.000 | | | | | | |
| Pseudo R2 | 0.039 | | | | | | |
| Mean Variance inflation factor | 1.58 | | | | | | |
| Mean Pregibon dbeta | 0.275 | | | | | | |
| Specification error (linktest): predicted value (_hat)[p>\|z\|] | 0.000 | | | | | | |
| Specification error (linktest): predicted value squared (_hatsq) [p>\|z\|] | 0.141 | | | | | | |

Source: Authors’ computation from unit records of NSSO 75^th^ Round 2017-18

**Additional Table 3: Factors affecting catastrophic health expenditure at 10% threshold (CHE-10) in India’s elderly, 2017-18**

| Total | Odds Ratio | Std. Err. | z | p>z | 95% Conf. Interval | | VIF |
| --- | --- | --- | --- | --- | --- | --- | --- |
| **Age group (years, ref:60-69)** |  |  |  |  |  |  |  |
| 70-79 | 1.023 | 0.051 | 0.460 | 0.646 | 0.929 | 1.127 | 1.080 |
| 80 and above | 1.003 | 0.074 | 0.030 | 0.972 | 0.867 | 1.159 | 1.090 |
| **Place of Residence (ref: rural)** |  |  |  |  |  |  |  |
| Urban | 0.504 | 0.027 | -12.910 | 0.000 | 0.454 | 0.559 | 1.400 |
| **Gender (ref: male)** |  |  |  |  |  |  |  |
| Female | 0.785 | 0.040 | -4.770 | 0.000 | 0.710 | 0.867 | 1.300 |
| **Surviving Children (ref: no child)** |  |  |  |  |  |  |  |
| At least one child | 0.753 | 0.089 | -2.400 | 0.016 | 0.597 | 0.950 | 1.040 |
| **Social Groups (ref: ST)** |  |  |  |  |  |  |  |
| SC | 1.348 | 0.143 | 2.830 | 0.005 | 1.096 | 1.659 | 2.370 |
| OBC | 1.456 | 0.135 | 4.040 | 0.000 | 1.213 | 1.746 | 3.750 |
| General | 1.408 | 0.133 | 3.630 | 0.000 | 1.170 | 1.693 | 3.950 |
| **Education (ref: illiterate)** |  |  |  |  |  |  |  |
| Up to primary | 1.184 | 0.070 | 2.850 | 0.004 | 1.054 | 1.329 | 1.270 |
| Up to secondary | 1.397 | 0.094 | 4.940 | 0.000 | 1.224 | 1.595 | 1.460 |
| Above Secondary | 1.481 | 0.122 | 4.760 | 0.000 | 1.260 | 1.741 | 1.800 |
| **Household occupation (ref: self-employed)** |  |  |  |  |  |  |  |
| Regular Wages | 1.009 | 0.063 | 0.150 | 0.881 | 0.894 | 1.140 | 1.280 |
| Casual Labourer | 0.985 | 0.069 | -0.220 | 0.827 | 0.858 | 1.130 | 1.200 |
| **Economic quintile (ref: poorest)** |  |  |  |  |  |  |  |
| Poor | 0.625 | 0.050 | -5.860 | 0.000 | 0.534 | 0.731 | 1.670 |
| Middle | 0.557 | 0.043 | -7.660 | 0.000 | 0.480 | 0.647 | 1.830 |
| Rich | 0.469 | 0.036 | -9.870 | 0.000 | 0.404 | 0.545 | 1.960 |
| Richest | 0.357 | 0.028 | -13.300 | 0.000 | 0.307 | 0.416 | 2.400 |
| **Insurance coverage (ref: No)** |  |  |  |  |  |  |  |
| Yes | 0.519 | 0.027 | -12.410 | 0.000 | 0.468 | 0.576 | 1.050 |
| **Provider (ref: public)** |  |  |  |  |  |  |  |
| Private | 8.178 | 0.408 | 42.170 | 0.000 | 7.417 | 9.017 | 1.110 |
| **Economic independence (ref: independent)** |  |  |  |  |  |  |  |
| Dependent | 1.057 | 0.059 | 1.000 | 0.318 | 0.948 | 1.180 | 1.310 |
| **Living arrangement (ref: with spouse/family)** |  |  |  |  |  |  |  |
| Living alone | 2.066 | 0.258 | 5.800 | 0.000 | 1.616 | 2.640 | 1.150 |
| **Constant** | 0.560 | 0.089 | -3.660 | 0.000 | 0.410 | 0.764 |  |
| **Model Details** | | | | | | | |
| Log likelihood | -6115.6486 | | | | | | |
| Number of observations | 10801 | | | | | | |
| LR Chi2 (22) | 2655.74 | | | | | | |
| Prob>Chi2 | 0.000 | | | | | | |
| Pseudo R2 | 0.178 | | | | | | |
| Mean Variance inflation factor | 1.65 | | | | | | |
| Mean Pregibon dbeta | 0.45 | | | | | | |
| Specification error (linktest): predicted value (_hat)[p>\|z\|] | 0.000 | | | | | | |
| Specification error (linktest): predicted value squared (_hatsq) [p>\|z\|] | 0.369 | | | | | | |

Source: Authors’ computation from unit records of NSSO 75^th^ Round 2017-18

**Additional Table 4: Factors affecting catastrophic health expenditure at 25% threshold (CHE-25) in India’s elderly, 2017-18**

| Total | Odds Ratio | Std. Err. | z | p>z | 95% Conf. Interval | | VIF |
| --- | --- | --- | --- | --- | --- | --- | --- |
| **Age group (years, ref:60-69)** |  |  |  |  |  |  |  |
| 70-79 | 1.073 | 0.059 | 1.290 | 0.197 | 0.964 | 1.194 | 1.08 |
| 80 and above | 0.990 | 0.081 | -0.120 | 0.902 | 0.843 | 1.163 | 1.09 |
| **Place of Residence (ref: rural)** |  |  |  |  |  |  |  |
| Urban | 0.528 | 0.031 | -11.000 | 0.000 | 0.472 | 0.592 | 1.4 |
| **Gender (ref: male)** |  |  |  |  |  |  |  |
| Female | 0.784 | 0.045 | -4.270 | 0.000 | 0.701 | 0.876 | 1.3 |
| **Surviving Children (ref: no child)** |  |  |  |  |  |  |  |
| At least one child | 0.811 | 0.099 | -1.720 | 0.086 | 0.638 | 1.030 | 1.04 |
| **Social Groups (ref: ST)** |  |  |  |  |  |  |  |
| SC | 1.547 | 0.205 | 3.290 | 0.001 | 1.193 | 2.006 | 2.37 |
| OBC | 1.676 | 0.199 | 4.350 | 0.000 | 1.328 | 2.116 | 3.75 |
| General | 1.593 | 0.191 | 3.870 | 0.000 | 1.259 | 2.015 | 3.95 |
| **Education (ref: illiterate)** |  |  |  |  |  |  |  |
| Up to primary | 1.264 | 0.084 | 3.520 | 0.000 | 1.110 | 1.441 | 1.27 |
| Up to secondary | 1.498 | 0.111 | 5.460 | 0.000 | 1.296 | 1.732 | 1.46 |
| Above Secondary | 1.597 | 0.143 | 5.220 | 0.000 | 1.340 | 1.904 | 1.8 |
| **Household occupation (ref: self-employed)** |  |  |  |  |  |  |  |
| Regular Wages | 0.934 | 0.067 | -0.950 | 0.343 | 0.812 | 1.075 | 1.28 |
| Casual Labourer | 1.133 | 0.090 | 1.570 | 0.117 | 0.969 | 1.324 | 1.2 |
| **Economic quintile (ref: poorest)** |  |  |  |  |  |  |  |
| Poor | 0.625 | 0.055 | -5.340 | 0.000 | 0.526 | 0.742 | 1.67 |
| Middle | 0.585 | 0.048 | -6.470 | 0.000 | 0.497 | 0.688 | 1.83 |
| Rich | 0.507 | 0.042 | -8.220 | 0.000 | 0.431 | 0.596 | 1.96 |
| Richest | 0.403 | 0.034 | -10.930 | 0.000 | 0.342 | 0.474 | 2.4 |
| **Insurance coverage (ref: No)** |  |  |  |  |  |  |  |
| Yes | 0.571 | 0.035 | -9.160 | 0.000 | 0.507 | 0.644 | 1.05 |
| **Provider (ref: public)** |  |  |  |  |  |  |  |
| Private | 7.519 | 0.474 | 31.990 | 0.000 | 6.645 | 8.508 | 1.11 |
| **Economic independence (ref: independent)** |  |  |  |  |  |  |  |
| Dependent | 0.940 | 0.057 | -1.030 | 0.303 | 0.834 | 1.058 | 1.31 |
| **Living arrangement (ref: with spouse/family)** |  |  |  |  |  |  |  |
| Living alone | 2.039 | 0.261 | 5.550 | 0.000 | 1.585 | 2.621 | 1.15 |
| **Constant** | 0.138 | 0.025 | -10.860 | 0.000 | 0.097 | 0.197 |  |
| **Model Details** | | | | | | | |
| Log likelihood | -5128.930 | | | | | | |
| Number of observations | 10801 | | | | | | |
| LR Chi2 (22) | 1793.75 | | | | | | |
| Prob>Chi2 | 0.000 | | | | | | |
| Pseudo R2 | 0.149 | | | | | | |
| Mean Variance inflation factor | 1.65 | | | | | | |
| Mean Pregibon dbeta | 0.245 | | | | | | |
| Specification error (linktest): predicted value (_hat)[p>\|z\|] | 0000 | | | | | | |
| Specification error (linktest): predicted value squared (_hatsq) [p>\|z\|] | 0.314 | | | | | | |

Source: Authors’ computation from unit records of NSSO 75^th^ Round 2017-18

**Additional Table 5: Factors affecting ‘living alone’ in India’s elderly, 2017-18.**

| Total | Odds Ratio | Std. Err. | z | p>z | 95% Conf. Interval | | VIF |
| --- | --- | --- | --- | --- | --- | --- | --- |
| **Age group (years, ref:60-69)** |  |  |  |  |  |  |  |
| 70-79 | 1.018 | 0.087 | 0.210 | 0.833 | 0.860 | 1.205 | 1.05 |
| 80 and above | 0.856 | 0.118 | -1.130 | 0.257 | 0.654 | 1.121 | 1.05 |
| **Place of Residence (ref: rural)** |  |  |  |  |  |  |  |
| Urban | 0.855 | 0.076 | -1.760 | 0.078 | 0.718 | 1.018 | 1.39 |
| **Gender (ref: male)** |  |  |  |  |  |  |  |
| Female | 2.824 | 0.248 | 11.850 | 0.000 | 2.378 | 3.353 | 1.14 |
| **Surviving Children (ref: no child)** |  |  |  |  |  |  |  |
| At least one child | 0.239 | 0.025 | -13.450 | 0.000 | 0.194 | 0.295 | 1.02 |
| **Social Groups (ref: ST)** |  |  |  |  |  |  |  |
| SC | 1.279 | 0.218 | 1.440 | 0.149 | 0.916 | 1.785 | 2.23 |
| OBC | 1.219 | 0.186 | 1.300 | 0.195 | 0.904 | 1.644 | 3.24 |
| General | 1.220 | 0.190 | 1.280 | 0.201 | 0.899 | 1.654 | 3.42 |
| **Education (ref: illiterate)** |  |  |  |  |  |  |  |
| Up to primary | 0.734 | 0.075 | -3.030 | 0.002 | 0.601 | 0.896 | 1.23 |
| Up to secondary | 0.363 | 0.047 | -7.740 | 0.000 | 0.281 | 0.469 | 1.41 |
| Above Secondary | 0.342 | 0.048 | -7.690 | 0.000 | 0.260 | 0.450 | 1.64 |
| **Household occupation (ref: self-employed)** |  |  |  |  |  |  |  |
| Regular Wages | 0.490 | 0.143 | -2.450 | 0.014 | 0.277 | 0.867 | 1.23 |
| Casual Labourer | 3.010 | 0.471 | 7.040 | 0.000 | 2.215 | 4.091 | 1.18 |
| **Economic quintile (ref: poorest)** |  |  |  |  |  |  |  |
| Poor | 0.603 | 0.091 | -3.350 | 0.001 | 0.449 | 0.811 | 1.57 |
| Middle | 0.789 | 0.106 | -1.760 | 0.078 | 0.606 | 1.027 | 1.64 |
| Rich | 1.109 | 0.139 | 0.830 | 0.409 | 0.867 | 1.418 | 1.71 |
| Richest | 1.380 | 0.167 | 2.660 | 0.008 | 1.089 | 1.749 | 1.98 |
| **Constant** | 0.009 | 0.002 | -21.270 | 0.000 | 0.006 | 0.014 |  |
|  | | | | | | | |
| Log likelihood | -2938.437 | | | | | | |
| Number of observations | 42760 | | | | | | |
| LR Chi2 (18) | 2396.66 | | | | | | |
| Prob>Chi2 | 0.000 | | | | | | |
| Pseudo R2 | 0.2897 | | | | | | |
| Mean Variance inflation factor | 1.63 | | | | | | |
| Mean Pregibon dbeta | 0.019 | | | | | | |
| Specification error (linktest): predicted value (_hat)[p>\|z\|] | 0.000 | | | | | | |
| Specification error (linktest): predicted value squared (_hatsq) [p>\|z\|] | 0.387 | | | | | | |

Source: Authors’ computation from unit records of NSSO 75^th^ Round 2017-18

**Additional Table 6: Factors affecting economic dependence in India’s elderly, 2017-18.**

| Total | Odds Ratio | Std. Err. | z | p>z | 95% Conf. Interval | | VIF |
| --- | --- | --- | --- | --- | --- | --- | --- |
| **Age group (years, ref:60-69)** |  |  |  |  |  |  |  |
| 70-79 | 1.930 | 0.057 | 22.270 | 0.000 | 1.821 | 2.045 | 1.050 |
| 80 and above | 3.591 | 0.193 | 23.730 | 0.000 | 3.232 | 3.991 | 1.050 |
| **Place of Residence (ref: rural)** |  |  |  |  |  |  |  |
| Urban | 0.975 | 0.028 | -0.870 | 0.382 | 0.921 | 1.032 | 1.310 |
| **Gender (ref: male)** |  |  |  |  |  |  |  |
| Female | 10.130 | 0.314 | 74.670 | 0.000 | 9.533 | 10.765 | 1.140 |
| **Surviving Children (ref: no child)** |  |  |  |  |  |  |  |
| At least one child | 2.064 | 0.142 | 10.570 | 0.000 | 1.804 | 2.361 | 1.020 |
| **Social Groups (ref: ST)** |  |  |  |  |  |  |  |
| SC | 1.057 | 0.055 | 1.050 | 0.292 | 0.954 | 1.171 | 2.210 |
| OBC | 1.197 | 0.055 | 3.930 | 0.000 | 1.094 | 1.309 | 3.240 |
| General | 1.185 | 0.056 | 3.620 | 0.000 | 1.081 | 1.300 | 3.410 |
| **Education (ref: illiterate)** |  |  |  |  |  |  |  |
| Up to primary | 0.849 | 0.029 | -4.820 | 0.000 | 0.795 | 0.908 | 1.230 |
| Up to secondary | 0.543 | 0.019 | -17.070 | 0.000 | 0.507 | 0.583 | 1.390 |
| Above Secondary | 0.261 | 0.011 | -30.890 | 0.000 | 0.240 | 0.284 | 1.570 |
| **Economic quintile (ref: poorest)** |  |  |  |  |  |  |  |
| Poor | 0.958 | 0.040 | -1.030 | 0.304 | 0.883 | 1.040 | 1.570 |
| Middle | 0.896 | 0.037 | -2.700 | 0.007 | 0.827 | 0.970 | 1.630 |
| Rich | 0.803 | 0.033 | -5.340 | 0.000 | 0.741 | 0.871 | 1.690 |
| Richest | 0.706 | 0.029 | -8.470 | 0.000 | 0.651 | 0.765 | 1.930 |
| **Living arrangement (ref: with spouse/family)** |  |  |  |  |  |  |  |
| Living alone | 0.164 | 0.014 | -21.540 | 0.000 | 0.139 | 0.193 | 1.030 |
| **Constant** | 0.637 | 0.053 | -5.390 | 0.000 | 0.540 | 0.750 |  |
|  | | | | | | | |
| Log likelihood | -19561.532 | | | | | | |
| Number of observations | 42755 | | | | | | |
| LR Chi2 (18) | 12375.97 | | | | | | |
| Prob>Chi2 | 0.0000 | | | | | | |
| Pseudo R2 | 0.2403 | | | | | | |
| Mean Variance inflation factor | 1.620 | | | | | | |
| Mean Pregibon dbeta | 0.709 | | | | | | |
| Specification error (linktest): predicted value (_hat)[p>\|z\|] | 0.000 | | | | | | |
| Specification error (linktest): predicted value squared (_hatsq) [p>\|z\|] | 0.081 | | | | | | |

Source: Authors’ computation from unit records of NSSO 75^th^ Round 2017-18
